# Speech is not special… again

**DOI:** 10.3389/fpsyg.2014.00427

**Published:** 2014-06-03

**Authors:** Kathy M. Carbonell, Andrew J. Lotto

**Affiliations:** Department of Speech, Language and Hearing Sciences, University of ArizonaTucson, AZ, USA

**Keywords:** sensorimotor effects on perception, multisensory integration, speech perception, auditory processing, Motor Theory

## The “specialness” of speech

As is apparent from reading the first line of nearly any research or review article on speech, the task of perceiving speech sounds is complex and the ease with which humans acquire, produce and perceive these sounds is remarkable. Despite the growing appreciation for the complexity of the perception of music, speech perception remains the most amazing and poorly understood auditory (and, if we may be so bold, perceptual) accomplishments of humans. Over the years, there has been considerable debate on whether this achievement is the result of general perceptual/cognitive mechanisms or “special” processes dedicated to the mapping of speech acoustics to linguistic representations (for reviews see Trout, 2001; Diehl et al., 2004). The most familiar proposal of the “specialness” of speech perception is the various incarnations of the Motor Theory of speech proposed by Liberman et al. (1967; Liberman and Mattingly, 1985, 1989). Given the status of research into audition in the 1950s and 1960s, it is not surprising that speech appeared to require processing not available in “normal” hearing. Much of the work at the time used relatively simple tones and noises to get at the basic psychoacoustics underlying the perception of pitch and loudness (though some researchers like Harvey Fletcher were also working on some basics of speech perception, Fletcher and Galt, 1950; Allen, 1996). Liberman and his collaborators discovered that the discrimination of acoustic changes in speech sounds did not look like the psychoacoustic measures of discrimination for pitch and loudness. Instead of following a Weber or Fechner law, the discrimination function had a peak near the categorization boundary between contrasting phonemes—a pattern of perceptual results that is referred to as Categorical Perception (Liberman et al., 1957). In addition, the acoustic cues to phonemic identity were not readily apparent with similar spectral patterns resulting in different phonemic percepts and acoustically disparate patterns resulting in identical phonemic percepts—the problem of “lack of invariance” (e.g., Liberman et al., 1952). The perception of these varying acoustic patterns was highly context-sensitive to preceding and following phonetic content in ways that appeared specific to the communicative constraints of speech and not applicable to the perception of other sounds—as in demonstrations of perceptual compensation for coarticulation, speaking rate normalization and talker normalization (e.g., Ladefoged and Broadbent, 1957; Miller and Liberman, 1979; Mann, 1980).

One major source of evidence in favor of a Motor Theory account of speech perception is that information about a speaker's production (anatomy or kinematics) from non-auditory sources can affect phonetic perception. The famed McGurk effect (McGurk and MacDonald, 1976), in which visual presentation of a talker can alter the auditory phonetic percept, is taken as evidence that listeners are integrating information about production from this secondary source. Fowler and Deckle (1991) have demonstrated a similar effect using haptic information gathered by touching the speaker's face (see also Sato et al., 2010). Gick and Derrick (2009) reported that perception of consonant—vowel tokens in noise are biased toward voiceless stops (e.g., /pa/) when they are accompanied by a small burst of air on the skin of the listener, which could be interpreted as the aspiration that would more likely accompany the release of a voiceless stop.

In addition, there have been several studies that have demonstrated that manipulations of the *listener's* articulators can affect perception, which are supportive of the Motor Theory proposal that the mechanisms of production underlie the perception of speech. For example, Ito et al. (2009) obtained shifts in phoneme categorization resulting from external manipulation of the skin around the *listener's* mouth in ways that would correspond to the deformations typical of producing these speech sounds (see also Yeung and Werker, 2013 for a similar demonstration with infants). Recently, Mochida et al. (2013) found that the ability to categorize consonants can be influenced by the simultaneous silent production of these consonants. Typically, these studies are proffered as evidence for a direct role of speech motor processing in speech perception.

Independent of this proposed motor basis of perception, others have suggested the existence of a special speech or phonetic mode of perception based on evidence of neural and behavioral responses to the same stimuli being modulated by whether or not the listener believes the signal to be speech or non-speech (e.g., Tomiak et al., 1987; Vroomen and Baart, 2009; Stekelenburg and Vroomen, 2012).

## The “generality” of speech

Since the early work by Liberman and colleagues and the development of the Motor Theory, there has been a growing appreciation for the power of perceptual learning and the context-sensitive nature of auditory processing. Once one begins to study more complex sounds and perceptual behaviors, the distinction between speech and non-speech processing becomes less clear. So, for example, we now have many examples of non-speech sound categories that demonstrate the characteristics of Categorical Perception (Cutting et al., 1976; Harnad, 1990; Mirman et al., 2004). It also appears that general auditory learning mechanisms are capable of dealing with the lack of invariance problem in formation of categories. Birds can learn speech consonant categories with no obvious acoustic invariant cue (Kluender et al., 1987) and human listeners can readily learn non-speech categories that are similarly structured (Wade and Holt, 2005). Finally, non-speech analogs have been created that result in the same types of context effects earlier witnessed for speech categorization, such as “perceptual compensation for coarticulation” (Lotto and Kluender, 1998; Holt et al., 2000), “speaking rate normalization” (Pisoni et al., 1983; Diehl and Walsh, 1989) and “talker normalization” (Watkins and Makin, 1994; Holt, 2005; Sjerps et al., 2011; Laing et al., 2012).

These findings with non-speech and animal perception of speech sounds (along with many others) call into question the strict dichotomy of speech and general auditory processing (Schouten, 1980). The lack of a clear distinction extends to the famed McGurk effect, which has been successfully modeled using general models of perception (e.g., Massaro, 1998). Stephens and Holt (2010) demonstrated that human adults can learn correlations between features of speech and arbitrary dynamic visual cues that are not related to the gestures of human vocal tracts. Participants in their experiments learned to associate the movements of dials and lighted bars on an animated “robot” display to stimuli varying in vowels and voiced consonant and could use this information to enhance intelligibility in noise. These types of novel mappings demonstrate the effectiveness of perceptual learning even across modalities (though perhaps not leading to as strong of an integration of information as may occur for natural covariations).

## The importance of research into multisensory interactions in speech perception

The growth in empirical research into the integration of multisensory information in speech acquisition and perception is a welcome development because it is a recognition that speech is not perceived within a vacuum. Too often, speech perception research has been conducted in an isolated reductionist vein that has made the human accomplishments in speech communication seem almost miraculous. The important realization at the heart of Lindblom's (1990, 1996) Hypo and Hyper Speech Theory is that much of the troubling acoustic variability in speech is actually a result of the changing demands of conversation between two people and the needs for informational precision due to the communication context. When one fails to study speech within a full communication context, this structured variability becomes noise. The isolation of speech research from a communication context has also made it difficult to connect the vast work in phonemic perception with more practical clinical issues in hearing loss and speech pathology. As Weismer and Martin (1992) point out, the concept of intelligibility must include both the speaker and the listener—that is, intelligibility is a measure of the entire communication setting and not just the acoustics of the speaker (see also, Liss, 2007).

The investigation of multisensory integration in speech perception is a step in the direction of attempting to understand the entire communication setting and all of the available information that results in an intelligible message. Some of the well-known findings from an auditory-isolated experiment may in fact be misleading when looked at in this broader context. For example, a highly cited finding is that 9-month-old infants from English-speaking households fail to discriminate a non-native Hindi contrast (Werker and Tees, 1984), which is taken as evidence that they are now perceptually tuned to their native language. However, Yeung and Werker (2009) obtained discrimination for infants in this group when the contrasting sounds were paired consistently with visual novel objects—a situation which mimics more realistically the communication setting of language learning. MacKenzie et al. (2013) in one experiment demonstrated an apparent unwillingness of 12-month-olds to associate novel auditory words with visual objects when the words are not phonotactically acceptable in their native language. However, the infants show far more flexibility in “acceptable” words when the task is preceded by a word-object association game with familiar word-objects. In each of these examples, the presumed perceptual tuning for language becomes less strict once the information available to the infant about the task is expanded. These experiments are stark reminders that speech acquisition and perception occurs in a larger perceptual/cognitive framework. Such results may also extend to adults learning to categorize speech sounds. Lim and Holt (2011) obtained significant increases in categorization performance for Japanese-speaking adults learning the non-native English /l/-/r/ distinction utilizing a video game paradigm. In this game, the categories were associated with different visual creatures that were either “friends” or “enemies” requiring different actions. The implicit mapping of auditory categories to functional dynamic visual objects may account for some of the success of this training.

## A cautionary note

Whereas the section above provides just a few of the many benefits of studying multisensory integration in speech, one must be cautious not to repeat the history of the field by proposing special mechanisms of phenomena for speech perception without thoroughly investigating what processes are available for general perception. The perception of all sound events is almost certainly intrinsically multisensory. Experimental designs that reduce sound event perception to audition run the risk of changing the task demands for the perceiver (as seen above in the examples for speech discrimination in infants).

There are many examples of sound perception being influenced by non-auditory information. Detection of low-intensity sounds is enhanced when paired with a task-irrelevant light stimulus (Lovelace et al., 2003; Odgaard et al., 2004). Saldaña and Rosenblum (1993) reported that when listeners were presented a visual image of a cello either being plucked or bowed, it strongly influenced their auditory judgment of whether the cello was being plucked or bowed. The perceived loudness of tones can be influenced by synchronous tactile information (Schürmann et al., 2004; Gillmeister and Eimer, 2007). In addition, sensori-motor interactions can be found in music perception (Maes et al., 2013). We should be very cautious in proposing multimodal or sensorimotor interactions that are “special” to speech. It is quite possible that new integrations between senses will be observed using the well-learned complex stimuli of speech sounds (or musical sounds) as opposed to simple noises and tones and unexperienced complex signals. These novel findings should be taken as opportunities to learn general principles of perception, action and cognition as opposed to assigning them special status and missing these opportunities.

Postulating a special speech perception mode or module is a strong theoretical position not to be taken lightly. One must describe how the processes brought to bear in the perception of speech sounds are fundamentally different from those responsible for other forms of complex audition. Speech sounds are “special” in the sense that they are over-learned categories that play a functional role in a larger hierarchical linguistic system. But these attributes on their own do not necessitate the proposal of inherently different processing mechanisms. In the end, speech sounds and the perception/categorization of these sounds is not likely to require special processing. The “specialness” of these sounds comes from being a part of the complex act of communicating. It is the act of communicating that clearly requires integration of the senses and the cooperation of perception and action. We must be wary that speech sound perception (“is this a “ba” or a”da”) isolated from the full act of communication is unnatural even when bringing to bear information from other sense modalities. The small and context-specific sensorimotor and multisensory effects we can uncover in this artificial task (Hickok et al., 2009) may not provide much insight into the real act of communication with speech.

### Conflict of interest statement

The authors declare that the research was conducted in the absence of any commercial or financial relationships that could be construed as a potential conflict of interest.
